# Breakage-Related Remanufacture of Mandibular Advancement Devices in Patients With Obstructive Sleep Apnea: A Retrospective Study of 219 Patients

**DOI:** 10.7759/cureus.117448

**Published:** 2026-10-04

**Authors:** Shintaro Kakuda, Shuto Ito, Yasuhisa Sawai, Yota Yamauchi, Fukunori Toukai, Satoshi Wada, Takanobu Takata, Eiji Mitate, Hiroyuki Nakano

**Affiliations:** 1 Department of Oral and Maxillofacial Surgery, Kanazawa Medical University, Uchinada, JPN; 2 Department of Physical and Cognitive Rehabilitation Technology, Kanazawa Medical University Hospital, Uchinada, JPN; 3 Department of Pathology Ⅱ, Kanazawa Medical University, Uchinada, JPN

**Keywords:** durability, mandibular advancement device, obstructive sleep apnea, oral appliance therapy, remanufacture

## Abstract

Introduction: Mandibular advancement devices (MADs) are used for obstructive sleep apnea (OSA), but the frequency of new-device fabrication following breakage during treatment is uncertain.

Methods: We analyzed 219 patients treated with a custom-made fixed-type MAD at a single center. The endpoint was the first documented new-device fabrication due to device breakage, across successive appliances if necessary, measured from the initial recorded fabrication date. The record term “repair” denoted new-device fabrication, whereas adjustment of an existing device was not an event. Dental-treatment and loss-related replacements did not end patient-level observation. For patients without a documented breakage event, absence of a return visit was assumed to mean no recorded event through March 31, 2025.

Results: Fifty-eight of 219 patients (26.5%) had a first breakage-related remanufacture; 161 were administratively censored. Two patients had an intervening new device for a non-device reason: one later had a breakage event and one did not. Median analytic observation time was 45.9 months (IQR 18.4-77.3; range 0.3-180.7). Estimated event-free survival at one and three years was 91.6% (95% CI 87.0-94.6) and 81.1% (95% CI 74.8-86.0), respectively. Actual last-contact dates and loss to clinical follow-up could not be determined.

Conclusion: Breakage-related new-device fabrication was documented during MAD treatment. These patient-level estimates depend on an unverified assumption that patients who did not return had no recorded event; they do not describe the physical lifespan of a single appliance or treatment efficacy.

## Introduction

Obstructive sleep apnea (OSA) is a common disorder characterized by recurrent upper-airway obstruction during sleep and is associated with impaired daytime function and cardiometabolic morbidity [1,2]. Positive airway pressure is an established treatment, while clinical practice guidance supports oral appliance therapy for appropriate adults, including those who prefer an alternative or cannot tolerate positive airway pressure [3]. Mandibular advancement devices (MADs) hold the mandible forward during sleep and can improve respiratory outcomes [4,5]. Long-term studies have also documented dental and occlusal changes during MAD treatment [6-10]. Evidence from clinical studies comparing treatment settings, appliance designs, and mandibular positions further shows that response varies among individuals and devices [11-17]. Treatment may continue for many years, making both therapeutic response and the condition of the appliance relevant to ongoing care. Professional guidance recommends dental oversight during oral appliance treatment to address dental changes and device-related problems [3].

Research on long-term MAD treatment has largely emphasized respiratory efficacy, adherence, and changes in the teeth and occlusion [4-10,18-23]. These outcomes do not directly indicate when a new device is needed because an appliance has deteriorated. Technical-complication studies offer useful context but may combine adjustments, repairs, and relines of an existing appliance with other maintenance procedures [24]. Such procedures have a different clinical meaning from fabricating a completely new MAD. Moreover, a new appliance may be required because a crown, extraction, or prosthetic treatment alters fit, or because the device is lost, even when its material has not failed. Counting all new appliances together would therefore obscure the frequency of remanufacture specifically attributable to appliance deterioration.

A reproducible assessment of breakage-related remanufacture requires a defined reason for fabrication, a date, and a distinction between making a new device and adjusting one already in use. In the present clinical records, the local term “repair” referred to fabrication of a new MAD, whereas “adjustment” referred to modification of the existing appliance. We analyzed the first documented new-device fabrication caused by breakage or clinically significant deterioration in each patient. Observation continued across a new appliance made for a non-device reason, such as dental treatment or accidental loss. Our objective was to estimate the time from initial recorded MAD fabrication to this first patient-level breakage-related event in a retrospective cohort of patients with OSA. This outcome concerns documented remanufacture during a patient’s treatment course; it does not estimate the physical lifespan of a single appliance or the effectiveness of OSA treatment.

## Materials and methods

Design and participants

This retrospective analysis used an existing cohort of 219 patients with OSA identified from records at Kanazawa Medical University Hospital from April 1, 2015, to March 31, 2025. Device histories predating April 2015 were retained, so cohort identification and historical device observation were not from the same period. The numbers screened and excluded before cohort assembly were unavailable. All 219 cohort records were analyzed. A sleep physician diagnosed OSA using polysomnography or out-of-center sleep testing.

The sample comprised available records, with no a priori sample-size calculation. Fifty-eight events constrain the precision of the survival estimates and any assessment of independent predictors. Confidence intervals are reported; post hoc observed power was not calculated. We did not undertake an adjusted Cox model because this retrospective prevalent cohort has incomplete exposure ascertainment, inconsistent observation of candidate determinants, and missing baseline covariates; these limitations preclude a credible causal predictor analysis.

Appliance and clinical assessments

The fixed-type MAD was fabricated in-house using 1.5-mm thermoplastic sheets joined by autopolymerizing resin. Initial mandibular advancement was approximately 70% of maximum protrusion measured with a George Gauge (Great Lakes Dental Technologies, Tonawanda, NY, USA). This describes the initial setting, not a measured final therapeutic position. A uniform final protrusion or a systematic rule for reducing protrusion in patients with temporomandibular discomfort could not be verified from the available dataset.

The original clinical description indicates assessment of usability, fit, retention, and device condition at clinical visits, with visits after early review occurring periodically or at patient request. Individual visits were not standardized or completely retrievable in this analysis. A documented decision to make a new device due to a clinically unsuitable cracked, fractured, deformed, or nonretentive appliance was classified as breakage-related remanufacture; no engineering test or blinded independent adjudication was available. The dataset did not verify that a standardized nightly-use instruction was delivered to every patient, and adherence measurements were unavailable.

Primary endpoint and reproducible classification

The endpoint was the first documented fabrication of a new MAD because of appliance deterioration or breakage during the patient’s treatment course. Illustrative causes include a clinically consequential crack or fracture, deformation, appliance-related loss of retention, or documented surface deterioration requiring a new device; these were operational examples, and the available records did not reliably allow counts by physical subtype. Discoloration alone did not qualify. A new MAD solely because of a crown, restoration, extraction, prosthesis, or accidental loss was categorized as non-device remanufacture.

The local chart term “repair” (*shuri*) referred to construction of a new MAD and was coded as remanufacture according to its cause. “Adjustment” (*chosei*) referred to alteration of the existing MAD without new fabrication and was not an event. A non-device remanufacture did not remove the patient from the risk set; subsequent devices and dated breakage events were reviewed until the first qualifying event or the study cutoff.

Cohort and breakage records were reconciled at the patient level; 27 histories required investigator clarification of causes or dates. Five had mixed causes over the device history: four had breakage first, and one had dental-related replacement followed by a documented breakage event on January 20, 2015. Another patient had replacement after accidental loss, without a later documented breakage event. These two non-device replacements were retained in follow-up. Explicit adjustment-only entries were excluded from events. Classifications were retrospective and were not independently reviewed by multiple blinded assessors.

Time origin, follow-up, and censoring

Time zero was the original recorded MAD fabrication date, with the clinic receipt date used as a proxy if a separate fabrication date was not recorded; an investigator-confirmed earlier fabrication date took precedence. Individual fabrication-to-delivery intervals were unavailable and could not be quantified. Patient-level follow-up continued through replacement for dental treatment or accidental loss and across the next device. The first documented breakage-related new fabrication was the event. If none was recorded, administrative censoring was set to March 31, 2025 regardless of whether the patient returned to clinic. An adjustment alone did not end observation. Time was calculated in days from baseline to first event or cutoff.

This administrative end date represents the explicit assumption that a patient without a documented return or breakage had no recorded qualifying event through March 31, 2025; it is not a verified date of patient contact, use, or event-free clinical follow-up. Actual last-contact dates, attendance after non-device replacement, loss-to-follow-up counts, discontinuation reasons, and return-visit frequency were unavailable. A sensitivity analysis censoring at last confirmed contact could not be performed. Undocumented events among nonreturning patients could bias event-free survival upward.

Statistical analysis

Recorded characteristics are summarized by mean and sample standard deviation with nonmissing denominators. Event/censoring time is reported by median, IQR, and range and does not represent confirmed clinical follow-up. A year was 365.25 days and a month 365.25/12 days. Patient-level event-free survival was calculated by Kaplan-Meier methods, with pointwise 95% intervals from Greenwood variance and a log-log transformation; events precede censoring on tied dates. Risk sets are shown at years 1, 2, 3, and 5. Missing Body Mass Index (BMI, 21/219) and Apnea-Hypopnea Index (AHI, 7/219) were not imputed; sex and BMI are reported descriptively, and no adjusted or subgroup significance analyses were undertaken. Analyses used Python 3.12.14 (Python Software Foundation, Beaverton, OR, USA), NumPy 2.3.5, pandas 2.2.3, and SciPy 1.17.0. Reproducible code and de-identified analysis fields are available for editorial inspection subject to institutional data-sharing approval.

Ethics

The Institutional Review Board (IRB) of Kanazawa Medical University approved this retrospective review on February 26, 2026 (C240), after the March 31, 2025 end of the clinical-record period. Written consent was waived with an opt-out procedure. The investigators confirm that research data extraction and analysis for this retrospective study began after IRB approval.

## Results

Cohort and observed follow-up

There were 219 patients (146 men, 73 women), with a recorded mean age of 51.9 ± 14.9 years. AHI was available in 212 and BMI in 198 patients; means were 19.7 ± 15.8 events/h and 24.3 ± 4.0 kg/m², respectively. Median analytic time to event or administrative censoring was 45.9 months (IQR 18.4-77.3; range 0.3-180.7); this does not establish duration of actual clinical attendance. Table 1 presents characteristics by endpoint status without tests of association.

**Table 1:** Characteristics by endpoint status without tests of association.

Recorded characteristics	All patients	Event	Censored
Patients, n	219	58	161
Men/women	146/73	39/19	107/54
Age, years	51.9 ± 14.9 (n=219)	55.5 ± 14.2 (n=58)	50.6 ± 15.0 (n=161)
AHI, events/h	19.7 ± 15.8 (n=212)	19.4 ± 18.1 (n=55)	19.8 ± 15.0 (n=157)
BMI, kg/m²	24.3 ± 4.0 (n=198)	23.9 ± 4.0 (n=51)	24.4 ± 4.0 (n=147)

Thirty-two original fabrication dates preceded April 2015, and 11 first breakage-related remanufactures occurred before that date; these historical records were retained. Fifty-eight patients had a first documented breakage-related remanufacture (26.5%), and 161 were administratively censored on March 31, 2025 (73.5%). One dental-treatment replacement was followed by a later breakage event; one loss-related replacement had no later documented breakage and was censored at the study cutoff. These intervening replacements are not separate censoring categories. Table 2 and Figure 1 show patient disposition; the number lost to actual clinical follow-up remains unknown.

**Table 2:** Patient-level estimates of breakage-related remanufacture. The number lost to actual clinical follow-up remains unknown.

Outcome disposition	n (%)
First breakage-related remanufacture	58 (26.5%)
Administrative censoring at March 31, 2025	161 (73.5%)
Total	219 (100%)
Intervening dental-treatment remanufacture (later breakage event)	1 (included above)
Intervening loss-related remanufacture (no later recorded breakage)	1 (included above)
Confirmed loss to clinical follow-up	Unavailable

**Figure 1:**
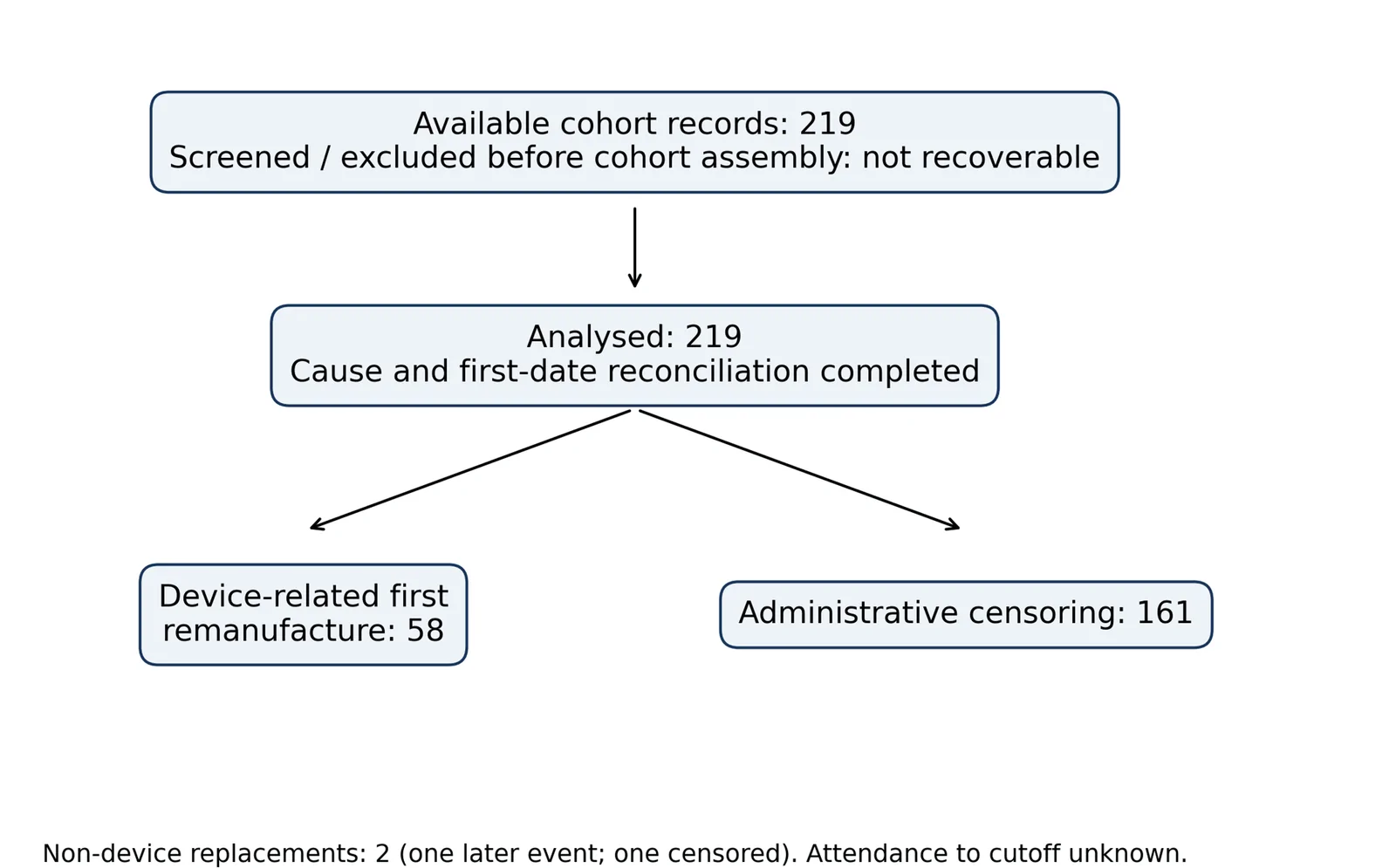
Available cohort disposition. The two intervening non-device remanufactures remain within the 219 patients. The cutoff assumes no documented qualifying event in nonreturning patients; clinic attendance to the cutoff cannot be confirmed.

Device-related remanufacture and survival

All 58 first events were classified as device breakage-related remanufacture after record reconciliation. Counts of cracks, fractures, deformation, appliance-related retention failure, or surface deterioration could not be reliably recovered. Table 3 and Figure 2 present Kaplan-Meier estimates with pointwise confidence intervals and risk sets. These results include first patient-level events after a prior non-device replacement but exclude later breakage events once a patient has already had a first qualifying breakage event.

**Table 3:** Kaplan-Meier estimates with pointwise confidence intervals and risk sets.

Years	Event-free survival, % (95% CI)	Number at risk
1	91.6 (87.0-94.6)	188
2	87.0 (81.6-90.9)	157
3	81.1 (74.8-86.0)	130
5	72.9 (65.4-79.0)	79

**Figure 2:**
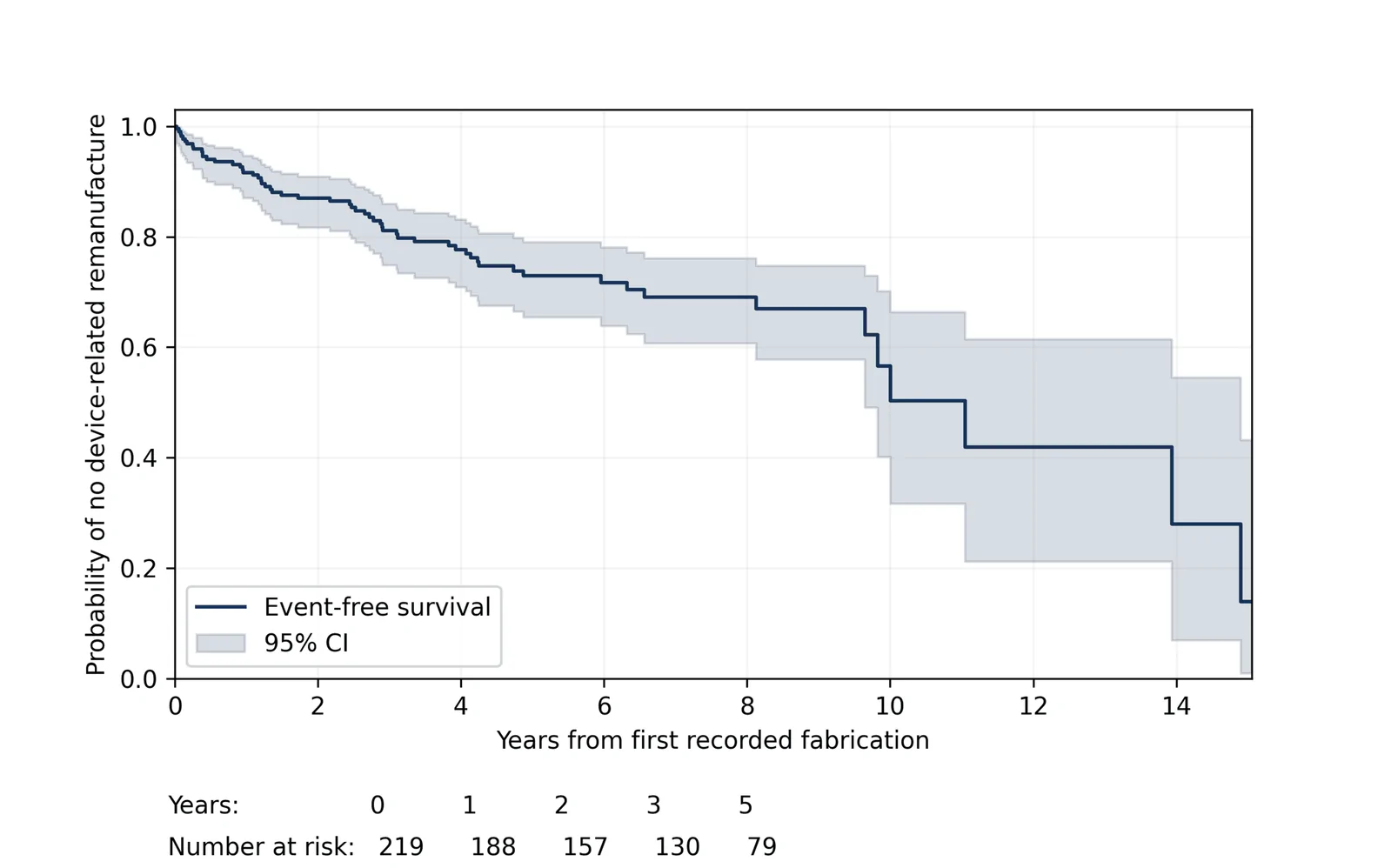
Kaplan-Meier freedom from a patient’s first documented breakage-related remanufacture, including successive MADs after non-device replacement. Shading indicates pointwise 95% confidence intervals. Administrative censoring on March 31, 2025, assumes the absence of an undocumented event. MAD, mandibular advancement device.

## Discussion

Fifty-eight of 219 patients (26.5%) had a first documented breakage-related remanufacture. Estimated three-year cumulative incidence under the study assumptions was 18.9% (95% CI 14.0-25.2%), calculated as one minus event-free survival. This patient-level measure combines exposure to successive appliances when a non-device replacement occurred. It cannot be interpreted as the physical lifespan of an initial appliance, the rate of all repeat fabrications, or treatment failure.

Studies of oral appliance treatment show improvements in OSA outcomes under various device designs and clinical conditions [5,11-17]. Such efficacy and adherence outcomes differ from our patient-level first breakage-related fabrication. A fixed-type design and initial advancement of approximately 70% cannot be generalized to other designs or titration schedules; final advancement and subsequent dose changes were not systematically measured.

Longitudinal dental studies report changes in occlusion and dentition during oral appliance therapy [6,8-10,18-23]. Such changes may affect fit without establishing a material defect. Conversely, a broken device can prompt remanufacture even when the patient has no documented change in respiratory outcomes. Our outcome categories therefore treat changes to teeth or prostheses and accidental loss separately from deterioration of appliance material or retention.

Martínez-Gomis et al. reported repairs, relines, and unscheduled visits in a five-year study of an adjustable MAD [24]. Their maintenance categories and attrition differ from the present new-fabrication event and patient-level observation across a non-device replacement. Direct comparisons of event rates are therefore inappropriate. Clinical records should distinguish an adjustment, maintenance of the same appliance, and fabrication of a new one.

This single-center retrospective prevalent cohort is subject to selection and survivor bias because device histories can precede cohort identification, and original fabrication dates were sometimes proxied by receipt dates. Unequal and patient-initiated visits create surveillance bias. Our assumption of no documented event in nonreturning patients is unverified; actual last contact and reasons for loss to clinical follow-up are missing, and occult breakage or treatment cessation may substantially raise the true event rate. Dental or loss-related replacement can alter exposure to subsequent devices. BMI and AHI are missing in some records, and sex, age, BMI, and AHI were not assessed as independent predictors. Bruxism, loading, dentition, fit, cleaning, adherence, actual advancement, and longitudinal OSA symptoms or objective sleep measures were not measured consistently. Confidence intervals quantify sampling error under the stated model, not these biases. STROBE reporting items were followed where records permitted [25].

## Conclusions

Breakage-related new-device fabrication was documented in 58 patients during MAD treatment. Clinicians should assess device fit and integrity when patients attend for care; this study does not establish an optimal surveillance schedule. The patient-level probability estimates depend on the completeness of event documentation and should not be interpreted as estimates of individual appliance durability or continued therapeutic effectiveness for OSA.
